# Delayed response to cold stress is characterized by successive metabolic shifts culminating in apple fruit peel necrosis

**DOI:** 10.1186/s12870-017-1030-6

**Published:** 2017-04-21

**Authors:** Nigel E. Gapper, Maarten L. A. T. M. Hertog, Jinwook Lee, David A. Buchanan, Rachel S. Leisso, Zhangjun Fei, Guiqin Qu, James J. Giovannoni, Jason W. Johnston, Robert J. Schaffer, Bart M. Nicolaï, James P. Mattheis, Christopher B. Watkins, David R. Rudell

**Affiliations:** 1 School of Plant Science, Horticulture Section, Cornell University, Ithaca, NY 14853 USA; 2 Boyce Thompson Institute for Plant Research, Cornell University, Ithaca, NY 14853 USA; 3 BIOSYST-MeBioS, KU Leuven, Heverlee, Belgium; 4 Tree Fruit Research Laboratory, United States Department of Agriculture, Agricultural Research Service, 1104 N. Western Ave, Wenatchee, WA 98801 USA; 5 United States Department of Agriculture, Agricultural Research Service, Plant, Soil, and Nutrition Laboratory, Ithaca, NY 14853 USA; 6 The New Zealand Institute for Plant and Food Research, Ltd, Havelock North, New Zealand; 7 The New Zealand Institute for Plant and Food Research, Ltd, Auckland, New Zealand; 8 Present addresses: AgroFresh, Wenatchee, WA 98801 USA; 9 Present addresses: Department of Horticultural Sciences, Mokpo National University, Muan, Korea

**Keywords:** Apple fruit, Chilling stress, Transcriptomics, Metabolomics, Cell death mechanism, Senescence, *Malus × domestica* Borkh

## Abstract

**Background:**

Superficial scald is a physiological disorder of apple fruit characterized by sunken, necrotic lesions appearing after prolonged cold storage, although initial injury occurs much earlier in the storage period.

To determine the degree to which the transition to cell death is an active process and specific metabolism involved, untargeted metabolic and transcriptomic profiling was used to follow metabolism of peel tissue over 180 d of cold storage.

**Results:**

The metabolome and transcriptome of peel destined to develop scald began to diverge from peel where scald was controlled using antioxidant (diphenylamine; DPA) or rendered insensitive to ethylene using 1-methylcyclopropene (1-MCP) beginning between 30 and 60 days of storage. Overall metabolic and transcriptomic shifts, representing multiple pathways and processes, occurred alongside α-farnesene oxidation and, later, methanol production alongside symptom development.

**Conclusions:**

Results indicate this form of peel necrosis is a product of an active metabolic transition involving multiple pathways triggered by chilling temperatures at cold storage inception rather than physical injury. Among multiple other pathways, enhanced methanol and methyl ester levels alongside upregulated pectin methylesterases are unique to peel that is developing scald symptoms similar to injury resulting from mechanical stress and herbivory in other plants.

**Electronic supplementary material:**

The online version of this article (doi:10.1186/s12870-017-1030-6) contains supplementary material, which is available to authorized users.

## Background

Superficial scald is a physiological disorder of apple (*Malus × domestica* Borkh.) fruit that results from necrosis of the first 5–6 cell layers of the apple peel [1, 2]. The disorder appears as relatively diffuse patches that sink with severity following 2–4 months of cold air storage or longer periods in controlled atmosphere (CA) storage. This disorder is a chilling injury associated with oxidative stress [3, 4]. A number of conditions, including harvest maturity and pre-storage light exposure all can mitigate scald incidence and severity. Cold temperature conditioning, intermittent warming periods, antioxidants, low oxygen storage, and inhibition of ethylene synthesis can eliminate or reduce scald severity [reviewed by 1]. Relationships among chilling stress, oxidative stress and ethylene action with scald etiology remain unclear as are the events that provoke and culminate in cell death many months following the start of cold storage. These relationships continue to be the focus of experimentation aimed towards better understanding metabolism and genomic controls of scald development.

Early targeted metabolic studies linked oxidation of α-farnesene, a prominent volatile component of ripe apples of many cultivars, and a variety of conjugated trienols and 6-methyl-5-hepten-2-one (MHO) with scald development [5, 6]. Inhibiting ethylene biosynthesis or action reduces CTOL and MHO biosynthesis production by reducing α-farnesene biosynthesis [7–9]. Application of the principal α-farnesene oxidation product, the 2,6,10-trimethyldodeca-2,7(*E*),9(*E*),11-tetraen-6-ol (CTOL) and other closely related compounds to apples at harvest resulted in symptoms visually similar to superficial scald and could be reduced in severity by prior DPA treatment [10]. However, beyond this evidence and in vivo [11] and in vitro [12] CTOL level reduction with DPA treatment, little evidence links CTOL generation with ROS generating metabolism within the fruit peel suggesting the process may coincide with scald provocation rather than be directly linked with it. The majority of earlier reports focus on gene expression related to α-farnesene synthesis given its established, albeit indirect, links with superficial scald. A 3-hydroxy-3-methylglutaryl-CoA reductase (*hmgr*2) [13], and α-farnesene synthase (AFS) gene expression can increase prior to or at the same time as α-farnesene production [14].

Chilling injury of chilling sensitive fruit is typically characterized as injury that results from rapid membrane phase separation leading to the immediate loss of membrane integrity and death [15]. Early evidence indicated that chilling-induced loss of mitochondrial [16] membrane integrity reduced oxidative activity differentially, effectively reducing respiration rate, depending upon tissue sensitivity to chilling temperatures. More recent reports regarding a wide scope of short-term fruit chilling injuries point to the involvement of ethylene and oxidative stress [17–19]. However, this type of short-term chilling injury is indicative of a catastrophic reaction to a low temperature event rather than a more controlled, or event directed, reaction whereas many apple fruit chilling injuries develop over weeks or, even months from the inception of cold storage as is in the case of superficial scald.

The degree to which cell death is controlled or “programmed” in the case of scald remains uncharacterized, although widespread metabolic changes occur during the storage period prior to disorder development [20, 21]. Little data about gene expression storage is available although a transcriptomic study of an cortex disorder of apples during CA storage revealed gene expression divergences prior to symptom development [22].

Systems approaches, incorporating two or more global data analysis tools, are increasingly employed to link experimental conditions and genotype with phenomic, proteomic, and transcriptomic changes. Metabolomic and transcriptomic evaluations enable comparison of unbiased snapshot of metabolism within a single sample for characterization and discovery of new areas of metabolism altered by a set of experimental conditions or linked with a particular phenotype [23–25]. Given the complexity of regulation and diversity of metabolites involved, these techniques have proven especially useful in numerous studies for the untargeted analysis of fruit ripening [26, 27]. Simultaneous transcriptomic and metabolomic evaluation has been used to find genes associated with tomato fruit color development in multiple genotypes [28] and to link ripening-related transcription factors with metabolites in a light sensitive tomato mutant during fruit ripening [29].

Metabolomic transitions during scald development are characterized by successive increases in concentration of classes of compounds during storage [20]. These increases are provoked by chilling stress and influenced by whatever storage conditions or treatments imposed until, finally, symptom development occurs and provokes its own metabolic shifts. Metabolic shifts under conditions that provoke or reduce scald development will define the chain of events and pathways specifically related to this form of chilling-related cell death and begin to establish whether this prolonged period culminating in cell death is an active process. Here, we have investigated scald development using a systems approach, incorporating untargeted metabolomic and transcriptomic evaluation to discover metabolic processes that are successively upregulated during a six month air storage period. It was expected that changes in this integrated global data set would precede symptom development in a coordinated manner indicative of the fruit tissues fate with respect to scald.

## Results

### Symptom development

**Fig. 1:**
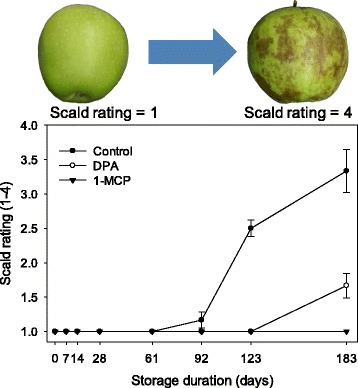
Scald severity (1 = no scald, 2 = less than 25%, 3 = 25–50%, 4 = greater than 75% coverage) on ‘Granny Smith’ apple fruit stored in air at 1 °C for up to 183 days (6 months). Apples were treated immediately following harvest with 2000 μL L^−1^ DPA or 1 mL L^−1^ 1-MCP. Error bars represent standard error (adapted from metadata presented in [20])

Tissue death resulting in scald was detected on control fruit between 2 and 4 months of storage. Injury symptoms reached between 50 and 75% months 6 (Fig. 1). Minor symptoms (<25% total coverage) appeared on DPA-treated fruit only at 6 months, while no symptoms developed on 1-MCP treated fruit.

### Untargeted metabolite analysis

Untargeted metabolic profiling provided a broad-based assessment of dynamic fluctuations within primary and secondary metabolism. Combining three extractions and four analysis protocols enabled the detection of free metabolites of wide-ranging polarity and volatility, including 162 identified and 432 partially characterized or unidentified metabolites (Additional file 1: Table S1). Identified metabolites included primary and secondary sugars, amino acids, and organic acids, photosystem pigments and co-factors, phenylpropanoids, phytosteryl membrane components, triterpenoids, volatile metabolites, lipids, triacylglycerols, oxidized sesquiterpenoids and triterpenoids and acylated terpenoids.

### Transcriptomic analysis

More than 630 million sequence reads were produced averaging 5.6 million per sample. Once rRNA and other contaminant sequences were removed, 84.7% (range 84–90%) of the cleaned reads aligned to the apple reference genome. If, for any gene, the sum of the three replications of any treatment/storage duration combination was greater than 5 RPKM (Reads Per Kilobase of transcript per Million mapped reads), then the gene was kept in the data set for further analysis. An additional 19 genes that were not detected at more than one treatment/storage duration combination were removed from this list of those that met the first criterion. 35,624 genes of the 63,541 physically annotated gene models met our criteria for consideration in further analyses (Additional file 1: Table S2).

### Summary of metabolomic-transcriptomic shifts during storage

Statistical analyses and data modeling were performed using multiple techniques to explore this large, complex, information-rich expression-metabolite data set. At first, it was established how metabolism was affected by known experimental inputs and phenotypic changes during storage. Then, the co-expression-metabolite network was evaluated as a whole, including fluctuations in gene expression or metabolite levels not directly linked with known inputs. Finally, sub-networks associated with processes that coincide or precede cell death were extracted from the larger network focusing on specific gene-metabolite linkages prior to functionally analyzing targeted gene models.

Principal components analysis (PCA) was used to create orthogonal data structures that summarize which experimental inputs provoked the greatest metabolic changes. For this analysis, transcriptomic and metabolomic data were interrogated separately. By differential positional changes over storage time among the different treatments, PCA scores (Additional file 2: Figure S1) illustrate divergent changes in the overall metabolome and transcriptome primarily associated with the initial chilling stress (those between 0 and 7 days), increasing storage duration, symptom development on control fruit. 1-MCP application reduced ethylene sensitivity and altered metabolism early in the storage period while DPA treatment had a lesser effect during this period. While changes in both of these data sets were primarily reflected in the first two principal components of both datasets, the total explained variance (33%, transcriptome; 51%, metabolome) was relatively small, indicating that other unidentified factors may contribute to variation of these datasets.

**Fig. 2:**
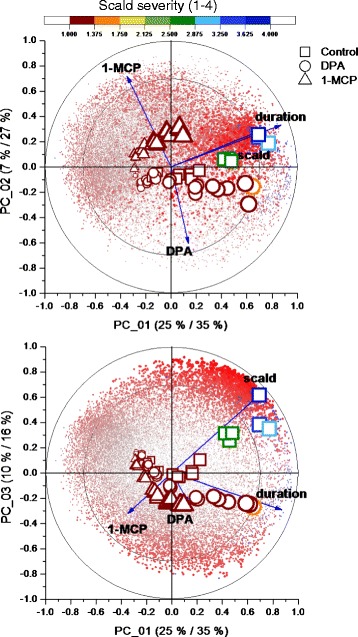
Multi-block Partial Least Squares Discriminate Analysis (MBPLS-DA) bi-plots of gene expression and metabolite level data from ‘Granny Smith’ apple peel from fruit stored in air at 1 °C for up to 183 days (6 months). Apples were treated immediately following harvest with 2000 μL L^−1^ DPA or 1 mL L^−1^ 1-MCP. PC1–2 (top) and PC1–3 (bottom) planes are represented. Shapes represent scores for each observation where symbol size increases with storage duration and symbol color represents scald severity. Emboldened text labels represent position of each response variable used for the model. Red points represent transcript and blue metabolite loadings where point size and opaqueness indicates each relative VIP score

After confirming the impact of known experimental inputs and phenotypic changes on overall metabolism, the effects of non-specific experimental variance were reduced using multiblock partial least squared discriminate analysis (MBPLS-DA). As with the unsupervised PCA analysis, most of the variance accounted for by MBPLS-DA was associated with change in the metabolome/transcriptome with storage duration as affected by DPA or 1-MCP treatment or the development of peel necrosis (Fig. 2). Following a sharp metabolic change over the first 7d of cold storage, metabolism of 1-MCP treated peel began to diverge from the control and then DPA treated peel following 28 d. Divergence between these latter two metabolomes/transcriptomes was most pronounced following 61 d or alongside the incidence and further progression of peel necrosis (Fig. 2, top). This divergence is particularly apparent when considering the PC1-PC3 plane where observations from control fruit beyond 2 months are clearly separate from the other treatments as are many transcripts and metabolites. The relationship between metabolites/gene models (x-variables) and experimental/phenotypic factors (y-variables) were ranked using each variables’ importance to the projection (VIP). The VIP estimates the importance of each variable in the projection of the MBPLS-DA model. A higher VIP score indicates a higher correlation among metabolites, genes, or other evaluated traits.

One striking change with storage duration was an increase in levels of a vast quantity of genes in 1-MCP treated peel as reflected in the scores plot. By contrast, levels of a small number of metabolites were elevated in these ethylene insensitive fruit over time while increases of metabolites had a relatively large impact on the overall changes in control and DPA treated metabolome/transcriptome and more so in control peel as necrosis spread. Expression levels of genes also had a large influence on the metabolic shifts highlighted by this model with levels of many transcript levels increasing with storage duration. Some of these were more associated with antioxidant-treated (healthy) peel, especially following necrosis of control peel which coincided with an increase in a different set of gene models. Our further analyses focused on determining those gene models and corresponding metabolomic changes associated with scald development of control peel.

### Assessment of scald-related VIPs

A total of 5486 genes were included in the scald symptom related VIP list. Of the 123 metabolites (Additional file 1: Table S1) on the VIP list, levels of 115 were positively correlated (VIP_metpos_) while 7 were negatively (VIP_metneg_) correlated with scald severity. Products of α-farnesene oxidation, including 2,6,10-trimethyldodeca-2,7 (E), 9(E), 11-tetraen-6-ol, the principal CTOL associated with conditions that lead to scald development was ranked relatively highly (# 171 of 610) but did not make the cutoff. This earliest evidence of metabolic oxidation is particularly important, although not accounted for using the multivariate protocol as it preceded scald symptom development, predominantly in control fruit, by 10 to 12 weeks. Therefore, they were not correlated with scald development, similar to the most highly ranked VIPs. VIP_metpos_ metabolites included another oxidized sesquiterpene and triterpenoids related to ursolic and oleanolic acids.

VIP_metpos_ also included acylated steryl glycosides (ASGs), a large group tentatively identified as acyl esters, diacyl- and triacylglycerides, two galactolipids (MDGD; 18:2,18:3 and 18:2, 18:2), *p*-coumaryl acyl esters and other possible membrane components. VIP_metneg_ included *p*-coumaryl acyl esters, both fully and partially identified, a partially characterized oxidized triterpenoid and another partially identified triterpenoid acyl ester, and another MDGD (18:3, 18:3). Taken as a whole, this overall VIP list is indicative of differential changes in membrane components during the transition to asymptomatic peel on protected fruit or scald on control fruit. Furthermore, elevated methanol and methyl ester production, may reveal transition of cell wall components specific to symptom development. Increasing levels of methanol and esters containing acetyl, 2-methylpropanoyl, propanoyl, butyryl, 2-methylbutyryl, pentanoyl, and hexanoyl acyl moieties coincided with scald symptom development. The potential role of elevated pectin methylesterase (PME) activity during scald symptom development, therefore, became a focus of the transcriptomic evaluation.

**Fig. 3:**
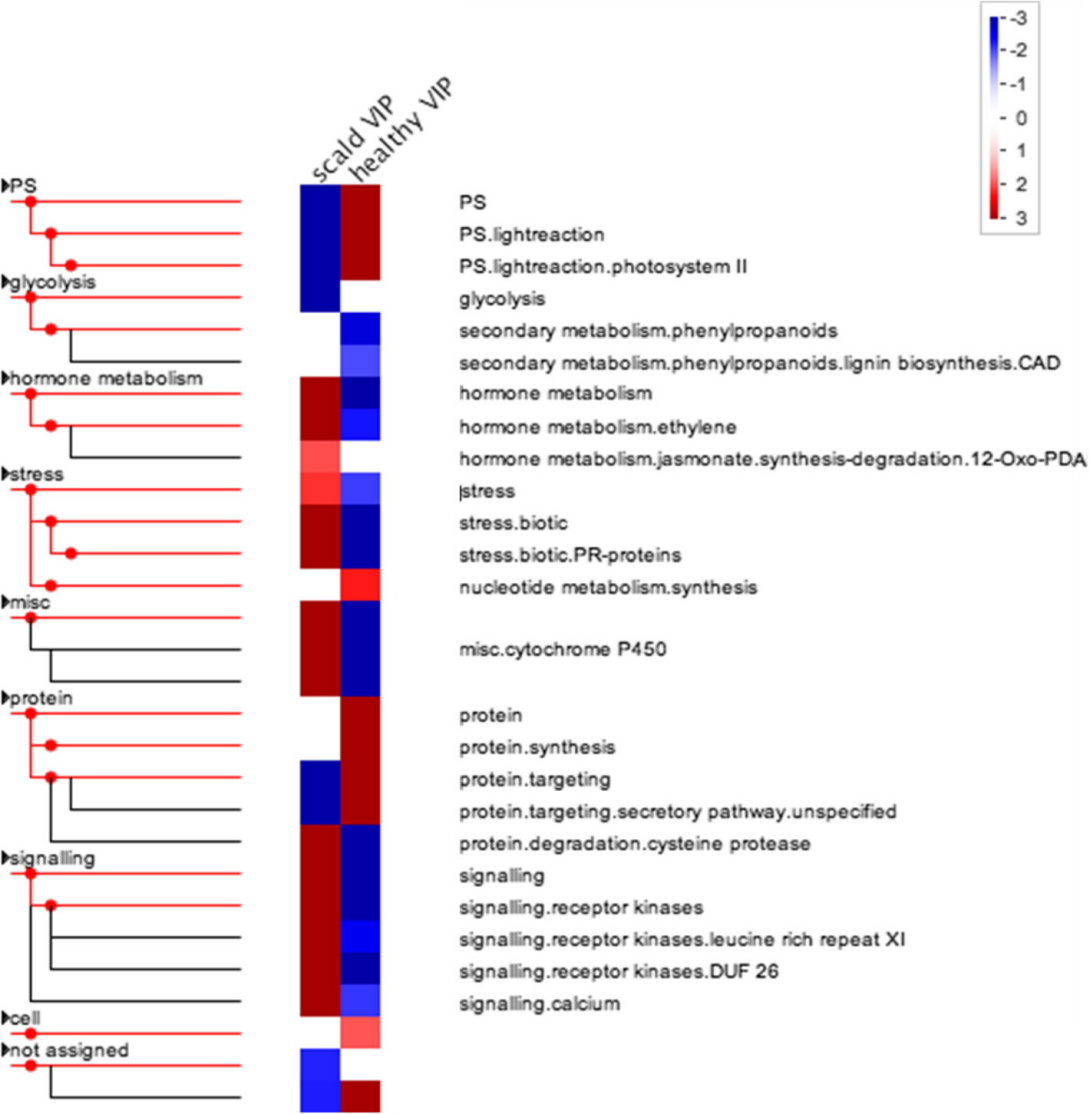
Pageman [92] over expression analysis of MBPLS-DA scald VIPs associated (scald; VIP_transpos_) or not associated (healthy; VIP_transneg_) with scald incidence. Red squares indicate relatively over expressed and blue under expressed categories within the scald-related VIP list. The Mdomestica_196 library and ORA_Fisher protocol were used form the analysis using Bonferroni correction and ORA cutoff = 1

Pageman overexpression analysis of 2724 gene model VIPs positively (VIP_transpos_) and 2762 negatively associated (VIP_transneg_) with scald symptom severity indicated differential changes in many processes associated with scald risk and/or eventual development (Fig. 3). Bin categories associated with stress were overexpressed in fruit that developed or would develop scald such as ethylene and jasmonate related metabolism, nucleotide metabolism, and receptor kinases and calcium signaling. Possibly more illuminating was over expression of genes related to energy production among the VIPs associated with healthy fruit (VIP_transneg_). Genes associated with photosynthesis were overexpressed in healthy fruit while under-expressed among scald associated VIPs. Similarly, glycolysis was under-expressed in fruit that was to develop scald. Protein synthesis related genes were overexpressed in healthy fruit while degradation related genes were overexpressed among scald associated VIPs.

### Correlation network analysis of phases preceding and coinciding with scald development

**Fig. 4:**
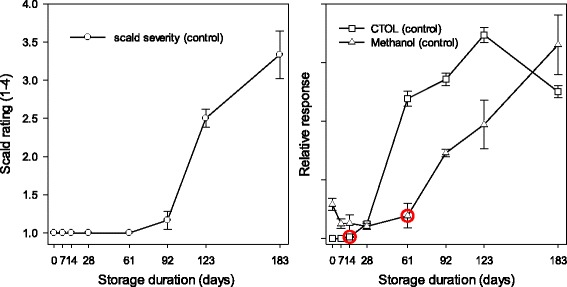
Superficial scald symptom severity (adapted from metadata presented in [20]), trimethyldodeca-2,7 (E), 9(E), 11-tetraen-6-ol (CTOL) levels and methanol levels in ‘Granny Smith’ apple peel sampled multiple times up to 183 days from air stored (0.5 °C) untreated (control) ‘Granny Smith’ apples. Red circles designate the time point at which increased production of CTOL or methanol began to increase. Error bars represent standard error

VIP ranking emphasized a wide range of metabolites and gene models directly associated with scald symptom development. More careful analysis of the dynamic fluctuations of levels of multiple metabolites known to be associated with scald or included in VIP_metpos_ revealed that a network analysis approach would provide further information regarding scald-risk specific changes prior to and during symptom development. To accomplish this, two phases of transcriptomic fluctuation were defined using changes in levels of CTOL and methanol (Fig. 4). CTOL began to increase between 2 and 4 weeks following storage inception or approximately 10–12 weeks prior to the first appearance of scald symptoms on control fruit. Methanol production began to increase between 8 and 12 weeks and the increase largely mirrored scald symptom development. Production of both CTOL and methanol were greatly reduced by DPA or 1-MCP treatment (data not shown).

**Fig. 5:**
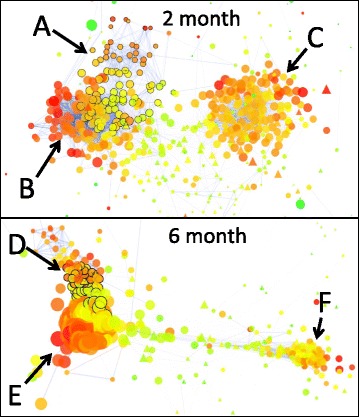
Undirected, edge-weighted gene expression/metabolite correlation networks generated from the first 2 months data (top) and all 6 months (bottom). Correlations R^2^ ≥ 0.700 were considered for network generation. Only nodes connected with 1 or more other nodes are included and, to highlight areas of connection, edges become more transparent as they diminish towards R^2^ = 0.700. Circles and triangles represent metabolite and gene clusters, respectively. Node size indicates neighborhood connectivity; larger nodes are connected to more closely correlated neighbors. Node color indicates clustering coefficient with *green* = 0, *yellow* = 0.64, and *red* = 1. Letters designate regions of interest including first neighbors (R^2^ ≥ 0.700) of CTOL—pre-symptomatic (**a**) or methanol-- symptomatic (**d**) networks with black outlined nodes, metabolites and transcripts linked with tissue that will develop healthy (**b** and **e**), and metabolites and transcripts with relatively elevated levels in unripe peel (**c** and **f**)

In our model, changes correlated with CTOL levels during the first 2 months represented increasing oxidative stress or initial responses to oxidative stress whether directly or indirectly related to fluctuations in gene expression or other metabolites and were the earliest metabolic evidence of the impact of oxidative stress associated with scald during storage. Increases occurring alongside methanol levels represented symptom-related or responses to symptom development. To find gene models associated with these two events, gene expression was, first, summarized using k-means clustering and two Pearson’s pairwise co-expression/metabolite networks were generated representing the first two months (Fig. 5, top) and representing all 6 months ( Fig. 5, bottom).

Clustering gene models established groups of fairly uniform gene expression patterns for correlation of average cluster behavior with individual metabolites. A total of 86 gene clusters contained varied numbers of gene models, averaging 414 per cluster ranging from 210 to 1275 (included in cluster 33) (Additional file 3: Table S3). Undirected, edge-weighted networks coupled with topological overlays highlighted metabolic complexes during storage indicative of healthy and pre-symptomatic (CTOL network) peel after the first 2 months of air storage (Fig. 5, top) or healthy and symptomatic peel (methanol network) after 6 months (Fig. 5, bottom) similar to those observed in the multivariate models. Metabolites and gene model clusters associated with unripe peel formed a third complex in both networks.

The subnetwork comprised of genes and metabolites that were first-neighbors with CTOL highlighted in the 2 month network ( Fig. 5, top) contained many metabolites also represented in VIP_metpos_, while other metabolites in VIP_metpos_, especially those most closely linked with symptom development, were not found within this group prior to symptom development. Other first neighbors in this list also included several direct and indirect α-farnesene and unknown triterpene oxidation products. Gene cluster 25 was the only gene model cluster neighboring CTOL. The subnetwork derived from first neighbors of methanol highlighted in the 6 month network (Fig. 5, bottom) was comprised primarily of highly-ranked scald-associated VIPs by the MBPLS-DA, although, unlike the 2 month network, it also contained compounds that closely co-fluctuate with scald symptom development. Methanol and acyl esters of methanol were among those compounds absent from the 2 month model yet present in the 6 month model. First-neighbors of methanol included methyl acetate, methyl propionate, methyl 2-methylproprionate, methyl butyrate, methyl 2-methylbutyrate, and methyl hexanoate as well as 3 gene model clusters (7, 33, and 55).

**Fig. 6:**
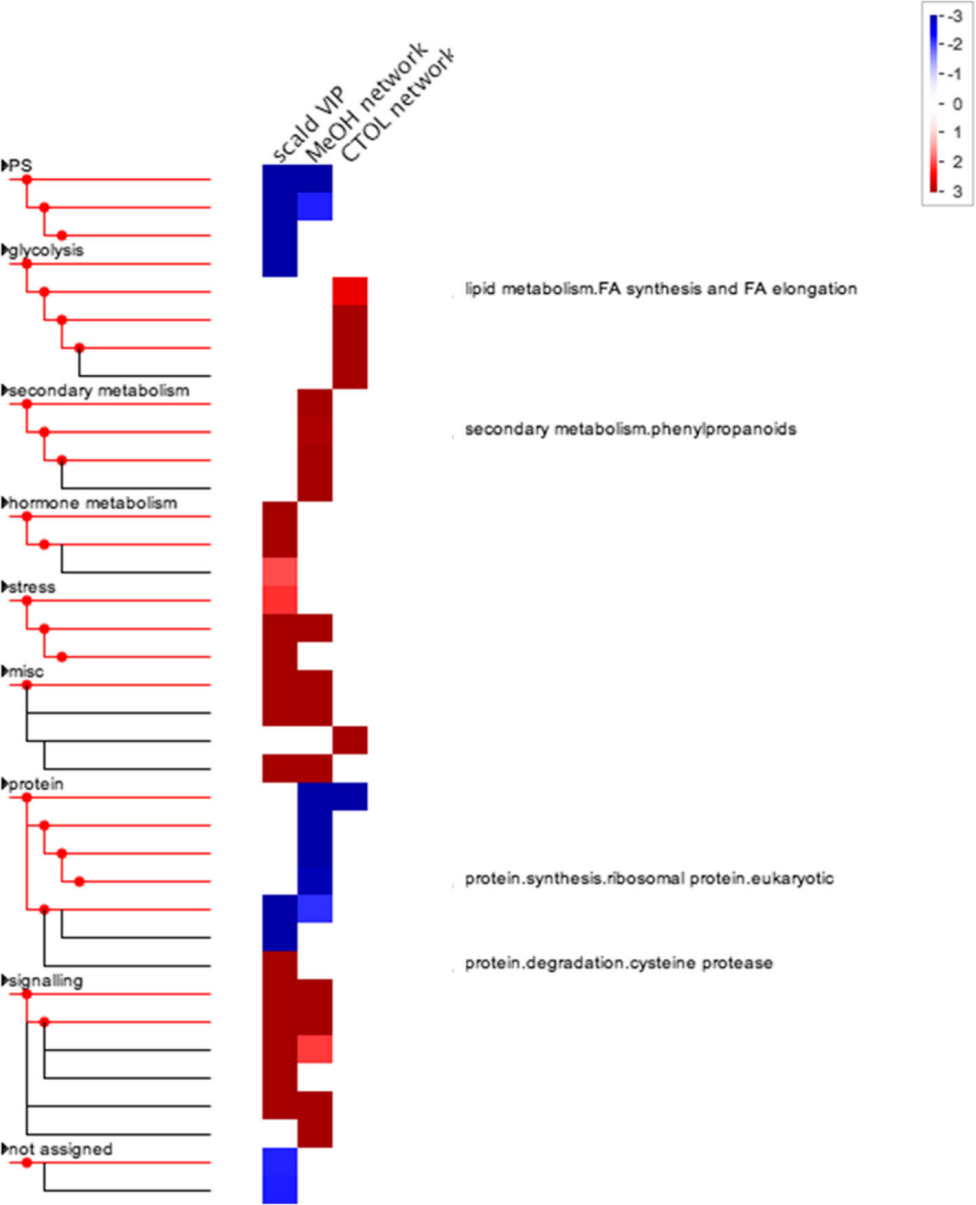
Pageman [92] over expression analysis of MBPLS-DA scald VIPs and genes contained in the “refined” methanol or CTOL sub-networks associated (scald) or not associated (healthy) with scald incidence. Red squares indicate relatively over expressed and blue under expressed categories The Mdomestica_196 library and ORA_Fisher protocol were used form the analysis using Bonferroni correction and ORA cutoff = 1

The k-means clustering protocol ultimately yielded many individual genes that were not as closely correlated with the CTOL (2 month network) or methanol (6 month network) levels as with the mean of the gene cluster where it was placed. Consequently, individual genes within gene model cluster 25 and clusters 7, 33, and 55 were refined to only those gene most correlated (R^2^ ≥ 0.700) with CTOL or methanol levels, respectively. Many, but not all, of the processes and genes over- or under-represented within the scald VIP list were similarly expressed in the methanol cluster (Fig. 6). However, the CTOL did not have similarly expressed processes given that it contained very few genes in common with those of the other categories. Genes involved in fatty acid synthesis and elongation were over-represented only in this network. Photosynthetic processes were under-represented both in the scald VIPs and the methanol network while various stress related and signaling processes were over-represented in both categories. Phenylpropanoid metabolism was over-represented and protein synthesis under-represented only in the methanol network. Hormone metabolism was only overexpressed in the scald VIP category.

### Identification of genes related to pectin methylesterase and ester volatile synthesis

**Fig. 7:**
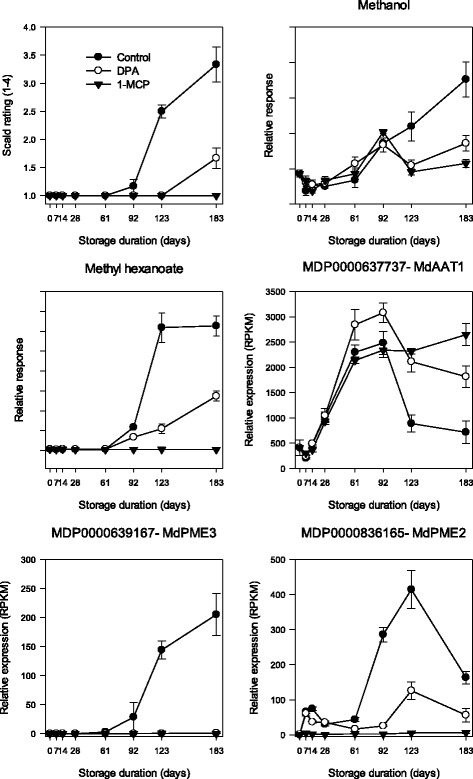
Methanol, methyl hexanoate, alcohol acyltransferase, and pectin methylesterase level in ‘Granny Smith’ apple peel from fruit stored in air at 1 °C for up to 183 days (6 months). Apples were treated immediately following harvest with 2000 μL L^−1^ DPA or 1 mL L^−1^ 1-MCP. Error bars represent standard error

Other genes were not represented in the overexpression analysis, yet may be linked by correlation and predicted catalytic function. These included cell wall process and number of pectin methylesterases (Additional file 4: Figure S2) whose expression correlated with methanol and acyl esters of methanol (Fig. 7). Gene models predicted to encode PME were compared with a *LePME1* (Solyc07g064170.2.1; partial AAB38794.1) that catalyzes demethylation producing methanol. MDP0000196922, MDP0000639167, and MDP000083616 (Additional file 5: Figure S3) within symptom associated gene model clusters were orthologs of LePME1 (Solyc07g064170.2.1). Along with *LePME1*, these paralogs included significant signature matches for the pectin esterase inhibitor (PF04043) domain and a PME (PF01095) domain comprised of a pectin lyase fold, and catalytic domains as well as the asp active site (Additional file 5: Figure S3). Expression of these LePME1 orthologs mirrored symptom development.

Alcohol acyl transferase (AAT) catalyzes the condensation of methanol with available acyl CoA components. Expression of *MdAAT1* (MDP0000637737) [30](AY707098) [31] or *MdAAT2* (MDP0000428199) [32] was similar, increasing immediately following storage imposition, and all were not affected by either DPA or 1-MCP treatment beyond reductions of expression from 3 to 6 months reflecting higher levels of symptom development (Fig. 7) opposite of the patterns of methanol and methyl ester production.

## Discussion

### Conditions associated with scald risk alter metabolome and transcriptome

An expectation of any parametric dataset is that the experimental conditions will impact the evaluated traits, especially where very large datasets are under interrogation and many of the latent factors yet require discovery [33]. Separate PCA of metabolomics and transcriptomic datasets provided evidence of divergence evoked during storage by oxidative stress and ethylene insensitivity (Additional file 2: Figure S1). Once the impact of these factors was established, supervision of these data with these same factors could be used to generate a MBPLS-DA model [34]. Integration of metabolomic and transcriptomic data sets into one model produced a more focused analysis by which individual metabolites and gene models could begin to be linked with their overall impact on actual scald incidence. MBPLS-DA afforded equal consideration of the transcriptome and the metabolome revealing a number of transcripts linked with scald development by further selecting only those predictor variables (metabolites and gene models) having the greatest impact on the projection (VIPs) when supervised by scald severity (a response variable) (Fig.  2).

### VIP list contains metabolites and genes most and least associated with symptom development

Determining which VIPs were enhanced with scald development yielded a list of metabolites, many already reported to be associated with scald, as well as methanol and methyl acyl esters, and gene models, including a number of transcription factors and PMEs. Other metabolites previously associated with scald risk include acylated steryl glycosides [21] as well as oleanolic and ursolic acid conjugates [20] Oxidized α-farnesene products, including CTOL, were associated with scald risk prior to scald development as indicated by many prior reports [1, 4, 20]. Because levels increase prior to and decrease as symptoms develop, VIP scores for these metabolites were relatively low and, consequently, they were not ranked highly enough to be included on the VIP list.

Where metabolic processes were either over- or under-represented in symptomatic fruit, the opposite was often observed in undamaged peel. Over representation of “plant stress” processes in peel that would eventually develop scald largely resulted from up-regulation of genes encoding pathogenesis-related (PR) proteins (Fig. 3). Expression of genes encoding PR proteins has long been linked both biotic and abiotic stress mediation, response, and, potentially, passive immunity in many plant species [35, 36] including those linked with chilling stress [37, 38]. Hypersensitive responses to infection or abiotic stress can provoke increased PR gene expression followed by patterned necrosis of leaves in a similar fashion to that observed in scald. Ethylene production and jasmonic acid accumulation are also common responses to chilling and other abiotic stressors in many plant species [39] and organs, including apple fruit [22, 40]. Ethylene signaling is integral to scald induction and development, especially given the complete inhibition of scald development with timely 1-MCP treatment [9, 41, 42]. While genes involved in jasmonic acid biosynthesis increase with apple ripening [43], altered concentrations have not been linked with wounding and cell death of apple. However, as with PR genes, jasmonic acid is associated with hypersensitive responses to multiple stressors in other plant species and organs [44].

Changes in pathways and processes associated with primary metabolism and energy production in peel that was developing scald is also a hallmark of stress response in plants, especially those associated with pathogen interactions [45]. Glycolysis was under-represented within VIP_tranpos_ and over-represented in VIP_tranneg_. Dynamic fluctuations of glycolytic metabolite levels were not linked with altered expression of glycolytic genes. Evaluating actual metabolic flux through the pathway is required to evaluate how stress may influence primary energy metabolism. Overall reduction of gene expression within primary metabolism of apple flesh as impacted by chilling stress coupled with high CO_2_ levels in storage has been reported [22]. Photosynthesis was similarly represented between the VIP categories, indicating that components of this process remain affected by chilling stress even in the absence of light. Chlorophyll content was altered as the tissue became necrotic during symptom development. Reduced expression of genes involved in photosynthesis during exposure to cold temperatures has been reported in *Arabidopsis* [46], which becomes increasingly less dramatic with sequential acclimation events [47]. Altered photosynthetic gene expression may be linked with changes in saturation [48] or constituency of phytosterol membrane components [21] during apple ripening and storage that are important for membrane fluidity and function in other plant species and tissues [49, 50].

### Co-expression networks reveal a scald risk related phase before symptom development

While the multivariate approach afforded confidence in our appraisal of scald-provoked transcriptomic changes by linking cold stress and scald with many expected areas of stress metabolism, it was limited by poor time resolution as it is only intended to account for major experimental impacts while trends over time represented by fewer variables that may be relevant to the sequence of metabolic events are absent from the first 3 dimensions of the model. A relatively simple correlation network was one way to both examine different phases of scald-associated metabolism and find individual genes from multiple processes that may be closely associated with the biosynthesis and regulation of specific scald-associated metabolites, specifically CTOL and MeOH.

Co-expression/metabolite fluctuation analysis was successfully employed to find trends that could be linked with scald yet had a relatively minor impact on the whole data set and, therefore, were undetected using multivariate analyses. Finding co-expression using pairwise correlation, essentially “guilt by association” [51] is the simplest approach to determining co-regulation among genes, although, realistically, any number of network motifs may indicate similar gene-gene regulation [52]. This approach has been successfully applied, alongside genetic and ontogenic contrasts, to identify genes associated with metabolite biosynthesis or distinct processes such as carotenoid biosynthesis [28] and transcriptional control of metabolism [29] during tomato fruit development ripening.

Our approach was to summarize gene expression and, then, correlate averaged k-means clusters with metabolite levels representing temporally different events linked with symptom development by treatment contrast (Fig. 5). We used this process to identify 2 groups of genes, representing gene expression in response to oxidative stress in the metabolome and, then later, changes primarily associated with injury development. For this analysis, our assumption, similar to that of Lee et al. [28] was that dynamic fluctuations of metabolite levels correspond with similar fluctuations of gene expression in some way related to the regulation or biosynthesis of that metabolite just as co-fluctuation of metabolite levels may indicate co-regulation. In this way, metabolites and genes with similar increasing expression trends during the pre-symptomatic stage are potential harbingers of impending disorder development.

The metabolic shifts occurring during storage may outline two of a series of events and responses beginning with initial scald related oxidation of the metabolome and culminating in scald development reported in the volatile signature by methanol and methyl ester production (Fig. 4). Temporally separated, multi-phasic responses are not uncommon in response to stress in plants. Biphasic responses to both abiotic stress and pathogen-elicited challenges can be comprised of altered stress-related metabolite levels or expression of stress associated genes [53]. Biphasic responses starting with elevated reactive oxygen species (ROS) levels and, then, ethylene production and related gene expression have been reported following pathogen challenge and subsequent defense or hypersensitive response (HR) [54–56].

Over-representation analysis of the CTOL and methanol networks in comparison with VIP_transpos_ was useful for indicating whether gene expression changes occurred starting with the first instance of metabolic stress or were more associated with symptom development (Fig. 6). For instance, under-representation of the photosynthetic process was evident in the methanol network but not the CTOL network indicating that the reduction of photosynthesis occurred alongside symptom development, or as the photosynthetic tissue died and not when stress was first evident in the metabolome. Phenylpropanoid metabolism was over-represented during this phase also and not among VIP_transpos_ or the CTOL network. Genes represented in this process included a polyphenol oxidase (PPO; MDP0000699845) reported to be associated with superficial scald in earlier studies [57, 58]. PPO expression can be induced by wounding and is associated with a loss of tonoplastic integrity and acidification of the cytoplasm resulting in polyphenol formation, a common response in plants to halt infection or wound proliferation [59]. The similar expression of three PMEs with its potential products within the 6 month network point to a possible role of these enzymes in this type of cell death and an atypical aroma profile.

### Superficial scald results from an active process

Intermittent warming, 1-MCP, and DPA treatments lose their effectiveness for scald control as timing of applications [3, 42] indicating that cumulative events that provoke the disorder transpire within this period in air storage. As HR typically leads to PCD, the etiology, symptom appearance, and, phasic response leading to scald development supports a view that scald may be a form of PCD distantly following cold stress. The protracted period from injury to symptom genesis associated with this disorder is a particularly compelling factor indicating at least some sort of resistance against the process or organized surrender for containment of the injury as it is in other systems [60, 61]. However, none of the principal hallmarks of plant PCD such as DNA fragmentation, protein digestion, or organized cytostructural events [60] have been reported for apple postharvest disorders. An early sub-cellular study of scald injury [62] provided clear evidence that loss of tonoplastic integrity, then other membrane structures, triggering phenolic oxidation in hypodermal cells occurred as symptom severity increased. Loss of membrane integrity, “blebbing”, and organelle leakage are all components of apoptotic cells. PCD related to postharvest fruit injury has been substantiated better in another instance. Cucumber fruit exhibit many hallmarks of PCD associated with continuous ethylene exposure such as enhanced endonuclease activity [63] and, subsequently, with chilling injury as demonstrated by DNA laddering and DNA lesions [64]. However, unlike superficial scald, chilling injury of cucumber typically occurs within a week following initiation of cold storage and at higher temperatures than those used for apple storage.

One clear outcome is that this correlative analysis highlights successive trends within this data set that can be traced to the impacts of ripening and oxidative stress as well as cell death that are not major factors in either PCA or PLSR analysis such as oxidation of a number of metabolites. A preponderance of evidence indicates scald is a chilling injury that occurs cumulatively within the first 1–2 months of air storage as DPA and 1-MCP treatments becomes successively less effective with increasing delay before treatment [42]. Consequently, the subsequent presence of oxidized metabolites may delineate the time period where resistance to oxidative stress is eventually compromised, leading to CTOL production and correlated gene expression which are the earliest indications of an irreversible cascade of events leading to cell death. Likewise, upregulation of numerous genes, including the three PMEs, and the unique volatile profile are active processes indicative of organization associated with symptom development supporting this possibility.

### Potential for PME as a conditional driver of methanol and methyl ester production

Methanol produced by fruit, including apple, can be a component of fruit aroma and is typically associated with ripening [65], although its genesis in apple fruit is uncharacterized. Apple alcohol acyl transferase (AAT) catalyzes condensation of alcohols and fatty acyl-CoAs producing esters [66]. While AAT gene expression and ester biosynthesis are both enhanced during ripening and as a result of ethylene exposure [66, 67], the capacity of both pre-climacteric and climacteric apples to immediately produce esters when exposed to alcohols, acids, aldehydes, and esters comprised of the moieties introduced [68–71], indicates the rate of alcohol acyl ester production by apple is, at least in part, controlled by the availability of alcohol and acyl-CoA substrate.

As expression of *MdAAT1* and *MdAAT2* remained largely unchanged, methyl ester production may be directly tied with methanol production catalyzed by specific PMEs (Fig. 7). Metabolism of both control and antioxidant treated apples in the current study was defined by a number of clearly ripening related processes such as enhanced overall volatile biosynthesis and diminishing levels of malic acid during the cold storage period while methanol and methyl ester production was only linked with symptom development, primarily from control fruit.

### Upregulation of PME and methanol production resulting from physical stress

In fruit, constituent PME catalyzes hydrolysis of homogalacturonan (HG) producing methanol and galacturonic acids [72, 73]. Enhanced PME expression and activity in many fleshy fruit [74], including apple [75], typically occurs during ripening as part of the softening process, although not necessarily associated with increased methanol production. However, methanol production can also result from mechanical stress that releases PME into the pectin-rich middle lamella during processing or juice production. Both LePME (partial, AAB38794.1; Solyc07g064170.2.1) activity [76] and, consequently, methanol [77] production were reduced in intact as well as following maceration in antisense LePME ‘Rutgers’ tomato fruit confirming the origination of methanol in tomato. The alignment and domain structure similarities among *LePME1* and its apple orthologs, MDP0000196922, MDP0000639167, and MDP000083616, which co-express with increasing methanol levels in symptomatic apple, support the likelihood of similar function warranting further functional analyses of the actual proteins (Fig. 7 and Additional file 5: Figure S3). Methanol production and PME expression also increases with mechanical damage in *Succisa pratensis* leaves [78] and, additionally, insect herbivory in *Nicotiana attenuata* leaves [79]. PME expression and methanol production provoked by insect feeding was eliminated in anti-sense PME constructs of *Nicotiana attenuata* [80].

Chilling and ethylene also enhance PME expression in plants [81]. Apple fruit with bitter-pit, a ripening and calcium related disorder, have elevated PME expression within tissue affected by the small corky lesions comprised of dead cortical tissue [82]. Bitter-pit symptoms develop prior to or during the initial phases following cold-storage inception and is not considered a chilling-related disorder like scald although intercellular calcium flux can have a role in programmed cell death [83] as suggested by de Freitas et al. [82] with respect to bitter pit. None of the genes evaluated by de Freitas et al. [82] were orthologs of the scald-associated gene models in this study [84].

While relationships between these events and PME expression remains undocumented, our results provided an indirect link with chilling and a direct link with wounding. A direct role for PME in cell death events remains to be clarified. PME may play a role in cell disassembly in a more coordinated programmed cell death event or even an attempt at cell wall stabilization by presenting active sites for Ca^2+^ binding and it is easy to see how these mechanisms may be linked in the cell death process in the case of bitter pit of apples. It has also been suggested that the resulting methanol production may be a signal of herbivory to neighboring flora in some plant systems [80]. Other than contributing to fruit aroma, although not commonly in most commercial apple cultivars, a physiological role of methanol presence remains to be determined in fruit. However, one practical outcome of apples producing methanol and methyl esters alongside scald development and, potentially other injuries, may be a signal that can be exploited by monitoring commercial apple storages for scald risk management in the absence of chemical treatments.

## Conclusions

Many of the factors that define scald, including the delay between cell damage and symptom appearance, and our evidence indicating shifting gene expression beginning with the first signs of oxidative stress of the metabolome, support the hypothesis that scald development is an active, organized process by which metabolism transitions towards cell death much as it does in the case of hypersensitive response to pathogen challenge or other established plant programmed cell death events. The culmination of symptom development also provides its own very specific metabolic and transcriptional profile including a unique volatile profile containing methanol and methyl esters compared to healthy, ethylene-receptive tissue. That the evolution of this scald aroma profile coincides with, and is likely a product of, PME activity links the disorder with cell disruption caused by physical maceration and resulting methanol production observed during tomato fruit processing and herbivory of other plants. Practical outcomes of this work include identification of processes, such as programmed cell death, that can be the focus of further work to establish genetic factors that predispose apples and other fruit crops to these types of chilling sensitivities. Also, the unique profile evolved from scalding or scalded tissue versus healthy tissues highlights opportunities for storage management practices that indicate risk of disorder development or disorder presence and afford fruit producers opportunities for mitigating losses by targeting fruit distribution and marketing. This work warrants further inspection programmed or controlled cell death caused by chilling stress of fruit as well as any roles that PME and methanol may have in cell death in plants.

## Methods

### Apple fruit harvest, treatment, and sampling

‘Granny Smith’ apples were harvested 140 d after full bloom from an experimental orchard located near Orondo, WA, USA and immediately transported to the laboratory, maturity was assessed and 1 μL L^−1^ 1-MCP applied as previously described [9]. Apples were treated with 2 g L^−1^ DPA as an aqueous emulsion containing DPA (Sigma-Aldrich, St. Louis, MO, USA) was formulated by dissolving 2 g DPA in a solution of 2 mL 2-propanol and 4 mL Triton X-100 (Sigma-Aldrich). Once dissolved, the solution was mixed with 1 L dH_2_O to form a milky emulsion. The same solution without DPA was used to treat control and 1-MCP treated fruit. To treat, fruit were submerged for 1 min, set on their calyx end, and allowed to air dry before sampling or placing them in cold storage.

Apples were stored in air at 1 °C for up to 6 months. Six replications of 3 fruit per treatment were removed from storage at 7, 14, 28, 61, 92, 123, and 183 d. Upon removal from storage, scald development was rated on a 0–4 scale (1, no scald; 2, 0–25%; 3, 25–50%; 4, >50% of the peel surface with symptoms; Fig. 1), peel sampled, and immediately flash frozen in clean N_2_ (*l*). Frozen peel was cryogenically milled to a fine powder using an N_2_ (*l*) cooled rotary mill (IKA Works, Willmington, NC). Samples were stored at −80 °C prior to metabolite and gene expression analysis. Using this procedure and storage protocol, the peel powder remained entirely friable.

### Metabolite extraction and analysis using GC-MS and LC-MS

Each frozen peel powder sample was analyzed using 3 extraction procedures and 4 instrumental analyses to evaluate the metabolome. Evaluation protocols included (described below) 1) abundant sugars and organic acid (TMS oxime dilute), 2) medium to low concentration polar metabolites (TMS oxime), 3) volatile metabolites (Volatile), and 4) non-polar metabolites (Non-polar).

**1 and 2) Trimethylsilyl (oxime) derivative analysis.** Methanolic extraction coupled with trimethylsilyl(oxime) derivatization and GC-MS analysis of frozen apple cortex powder (0.1 g) was carried out as previously described [85] with a modification to the TMS oxime dilute method as follows: The same sample was injected twice; for TMS oxime, a splitless inlet was maintained for the first 2 min of the run and for TMS oxime dilute, a 1:35 split was maintained for that period. Injection volumes were 0.2 μL for both analyses.

**3) Volatile metabolite analysis.** Volatile headspace analysis was performed using an automated Gerstel (Gerstel, Baltimore, MD) multipurpose sampler (MPS) equipped with a dynamic headspace sampler (DHS) and a Agilent 6890 N gas chromatograph coupled with a 5975B mass selective detector (Agilent Technologies, Palo Alto, CA) and as previously described [20].

**4) Non-polar metabolite analysis.** *Sample extraction.* Extraction and single quadrupole LC-MS analysis was performed as outlined in Rudell et al. [20].

*LC-MS Q-TOF.* Accurate masses for each compound were acquired by extracting samples as described above and introducing them into a 1260 Series Infinity HPLC coupled with a 6520 quadrupole-time of flight mass selective detector (Agilent Technologies, Santa Clara, CA, USA) and an APCI source. HPLC conditions were the same as outlined above. Detector conditions were as follows: drying gas (N_2_) flow 4 L min^−1^, drying gas temperature 350 °C, nebulizer pressure 414 kPa, vaporizer temperature 425 °C, coronal discharge of 4 μA, and fragmentor and capillary potentials 125 and 4000 V, respectively. The instrument was controlled using MassHunter Data Acquisition software (Agilent Technologies, Santa Clara, CA, USA). A reference solution containing purine (121.050873) and (1H, 1H, 3H–tetrafluoropropoxy) phosphazine (922.009798) (API-TOF Reference Mass Solution Kit) was continuously introduced into the source to assure mass accuracy remained <2 ppm for the analyses.

### Extraction and analysis quality control (QC)

Three reference samples were run daily with each GC-MS and LC-MS sequence to monitor the consistency of the extraction protocols and instrumental analyses. The reference material used for these samples consisted of a bulk sample of ‘Granny Smith’ peel obtained from fruit stored for 4 months in regular atmosphere at 1 °C, a storage condition and ontogeny that best assured that all of the metabolites detected were also detected in the QC standard. Compound levels of reference samples were assessed daily to ensure relative responses among reported metabolites remained similar across the entire experiment. Internal standard variation of less than 10% relative standard deviation within a sequence was considered acceptable and used as a criterion for rejection. If variation exceeded acceptable levels, samples from that day were re-extracted and analyzed.

### Data acquisition, deconvolution, and peak identification

To assure major metabolites were considered in our evaluation, existing in-house mass spectral libraries and metabolite lists were appended with metabolites found in 4 of 6 replications of 0, 61, and 183 d samples from control and DPA treated fruit. 1-MCP treated peel from 183 d was checked for unique compounds and none were found.

GC-MS and LC-MS libraries were generated using the automated mass spectral deconvolution and identification system (AMDIS; National Institute of Standards, Gaithersburg, MD, USA) to find unique components within the chromatographic mass spectral data [20]. For GC-MS data, AMDIS models for each component in samples were conserved for the libraries until, if considered, authentic standards were run and, then, those mass spectra would append the existing mass spectra in the AMDIS libraries. For LC-MS (single quadrupole) AMDIS was simply used as a visualization aide to find unique components and their mass spectral peaks. In both cases, target ions were selected based on the relative mass spectral peak height in comparison with other mass spectral peaks of that component and their uniqueness within that chromatographic neighborhood, where co-elution was an issue. Peak retention indices (RI) were generated for GC-MS data by calculating the Kovat’s index for each component based on a C10-C40 hydrocarbon standards evaluated with every sequence under the same conditions for comparison between the library and samples. Libraries were compiled and data analyzed as outlined by Rudell et al. [85].

### Chemical standards and identification

Identified, partially characterized, and unidentified metabolites are listed in Additional file 1: Table S1 alongside compound identification, partial identification, and retention index (GC) or retention time (LC). For LC-MS, mass spectral and/or accurate mass comparison with authentic standards were the bases for any confirmed identification. Tentative identifications were based on mass spectral or accurate mass similarity of target and other mass spectral peaks as well as fatty acid losses (TAGs, lipids, unknown triterpenyl, unknown farnesyl, and *p*-coumaryl conjugates) and similarity to published mass spectra (triterpenoids). Chemical standards were either purchased or obtained by a combination of synthesis or purification methods as designated in Additional file 1: Table S1 and Additional file 6: Protocol S1.

### RNA isolation and mRNA-seq library construction

Equal masses of frozen peel powder from replications 1 and 2, 3 and 4, and 5 and 6 (used for untargeted metabolic profiling) were compiled to create replications 1–3 creating 3 composite (6 fruit each) biological replications for each orchard/time point combination. RNA-seq libraries were generated using a method modified from Zhong et al., [86] where 22 libraries were multiplexed per reaction using an Illumina HiSeq 2000/2500 according to the protocol described by Gapper et al. [87].

### Bioinformatics

40 base pair single-end, strand-specific RNA-Seq reads were filtered by aligning rRNA and tRNA sequences to adapter using Bowtie [88], mapped using Tophat [89] and then normalized to reads per kilobase of exon model per million mapped reads (RPKM) as described by Gapper et al. [87].

### Multivariate analysis

Data collected from transcriptome and metabolome analyses were analyzed in Matlab (Matlab R2012b, The MathWorks, Inc., Natick, MA, USA) using the PLS-toolbox (v5.5.1, 2009, Eigenvector Research, Inc. Wenatchee, WA, USA), the Multi-block Toolbox (v0.2, 2004, University of Copenhagen, Denmark), in-house made MatLab procedures, and The Unscrambler X (v10.3, 2013, CAMO Software AS, Oslo, Norway).

For PCA and MBPLS-DA, data from each variable was individually normalized by mean centering and, then, standard deviation weighting. First, to establish the impacts of the employed experimental factors without supervising the analysis, PCAs (Unscrambler X) were, separately, applied to metabolomics and transcriptomic data providing an overview by projecting the multidimensional data on a smaller number of latent variables called principal components (PC). To prevent overshadowing the metabolomic data by the much larger transcriptomic dataset, MBPLS-DA modeling was chosen to maintain the ‘natural blocking’ within the dataset by considering relationships between the different blocks (metabolomic and transcriptomic data), in relation to their relative contribution to the treatment variables. In MBPLS-DA (Matlab, Multi-block tool box), each data block is considered separately with their common structure expressed through an additional top layer. The treatment variables are regressed on this super descriptor block. Object scores and variable loadings of the two underlying decompositions (one for the metabolome, one for the transcriptome) are optimized for object consensus at the super level. The super block-weights indicate how important each block of data is in defining the subsequent latent variables of the super descriptor block. For MBPLS-DA, genes and metabolites were used as independent x-variables while a categorical variable, indicating treatment, and continuous variables, indicating time and scald incidence, were used as dependent y-variables.

Variable Importance in Projection (VIP) scores were calculated according to Wold [90] as a weighted sum of squares of the PLS-weights, with the weights calculated from the amount of y-variance of each PLS component. The VIP estimates the importance of each variable in the projection used in a PLS model. The average VIP, by definition, equals one. In the MBPLS-DA context, the VIP was calculated similarly on each data block resulting in equal total contributions towards explaining each of the response variables. As the metabolome data contained 610 metabolites while the transcriptome data contained 36,253 gene models, the VIPs of the metabolites averaged 60 times higher compared with the transcripts. The rule that VIPs higher than 1 indicate important variables is therefore no longer valid in a MBPLS-DA context. For this reason it was decided to select the highest VIP values following*VIP* > *μ*_*VIP*_ + *t*_*p* , ∞_ ⋅ *σ*_*VIP*_with *p* ≤ 0.33 for the metabolites and the transcripts.

### Network creation and analysis

Gene expression data (RPKM) were summarized using k-means clustering (Matlab 2012b, The Mathworks, Inc., Natick, MA, USA) of the PCA smoothed expression data. To accomplish this, a PCA model was generated for the complete dataset and modeled values based on the first 10 PCs were used for clustering. While clustering was initialized using 100 random centroids, after deleting empty clusters, 86 gene clusters remained. The 2 month model was used to consider only correlations during that period as it eliminates the influence of decreasing levels of CTOL during the last two months associated with symptom development and captures all gene models that began to increase alongside CTOL. Subsequently, gene expression was averaged for each gene cluster and pairwise Pearson’s correlations with the individual metabolites were calculated either only for the first 2 months of data (2 month network) or the whole 6 months of data (6 month network). Only edges representing a R^2^ > |0.700| were included. The matrix was used to generate an undirected network model which was spatially represented using the edge weighted, spring embedded, edge-weighted layout to highlight regions of potentially similar regulation, or network neighborhoods, among metabolites and gene model clusters. Edges were weighted using 1-R^2^ (correlation coefficients) interpreted heuristically and all entered weights applied with a default edge weight = 0.5. The average iterations per node = 40, spring strength = 15, spring rest length = 45, strength of disconnected spring = 0.05, Rest strength of disconnected spring = 2, strength applied to avoid collisions = 0, and number of layout passes = 2. The generation of the graph was randomized and applied to all nodes. Network neighborhoods were further analyzed, defined, and illustrated using the “Network Analyzer” app [91] included with the Cytoscape package. Values used for the node size and color coding were “Neighborhood Connectivity” and “Clustering Coefficient”, respectively. Network connectivity is defined by the average connectivity of all neighbors of a certain node while clustering coefficient is the ratio of the actual number of edges to the maximum number of edges existing between neighbors of a specific node. As with the layout, these analyses are all targeted towards highlighting regions of similar regulation based on correlation and overall connectivity.

### Analysis of gene lists and apple pectin methylesterases

The k-means procedure used to summarize the gene expression data included outliers in each of the list. These genes were not as highly correlated with CTOL or methanol levels and, as the objective was to obtain lists of genes most highly correlated with trends in these metabolite levels, further refinement was required prior to further analysis of the genes within the clusters. To accomplish this, genes included in cluster 25 (correlated with CTOL levels) and clusters 7, 33, and 55 (correlated with methanol levels) were correlated individually with CTOL or methanol and any gene with R^2^ < 0.700 was removed from the respective list. Subsequently, overexpression analysis (ORA) gene VIP lists and members of the refined CTOL and methanol subnetworks was performed using PageMan [92], a component of the MapMan package, version 10 [93]) employing the Mdomestica_196 library. Gene lists were analyzed for over or under expression using the ORA_Fisher protocol with Bonferroni correction and an ORA cutoff of 1.0. To begin to quality function, scald-related pectin methylesterase genes were aligned and domains compared with orthologs from Arabidopsis (AT1G02810.1) and tomato (Solyc07g064170.2.1), the latter with known methanol production function using Geneious 6.0.

## Additional files

Additional file 1: Tables S1. Retention time/ retention index and target ion, partial or confirmed identification, and association with superficial scald risks of ‘Granny Smith’ apple peel metabolites analyzed using HPLC-MS and GC-MS. Table S2. Annotation and association with superficial scald of genes detected using RNA-seq of ‘Granny Smith’ apple peel. (XLSX 2108 kb)

Additional file 2: Figure S1. Principal Components Analysis (PCA) bi-plots of metabolite (top) and gene expression level data (bottom) from ‘Granny Smith’ apple peel from fruit stored in air at 1 °C for up to 183 days (6 months). Apples were treated immediately following harvest with 2000 μL L^−1^ DPA or 1 mL L^−1^ 1-MCP. Gene expression and metabolite datasets were analyzed separately. Shapes represent scores for each observation where symbol size increases with storage duration and symbol color represents scald severity. Gray points represent loadings for individual metabolites or transcripts. (DOCX 2373 kb)

Additional file 3: Table S3. Gene cluster assignment for correlation networks. To summarize gene expression data, genes expression patterns from all time points (0–183 d) and treatments (control, diphenylamine, and 1-methylcyclopropene) were clustered using k-means clustering. Average gene expression values from each cluster were correlated along with relative metabolite levels to generate 2 month and 6 month networks. The number of Variables Important in the Projection (VIP) for the “scald severity” response variable from the PLS-DA model is included for each gene cluster. (DOCX 18 kb)

Additional file 4: Figure S2. Comparative expression of scald VIPs and the “refined” methanol and CTOL subnetworks within different MapMan cell wall metabolism bins. Darker blue squares indicate elevated expression compared with the other lists within that category. (DOCX 29 kb)

Additional file 5: Figure S3. Alignment and domain comparison of scald-related MdPMEs, AT1G02810.1, and Solyc07g064170.2.1, a methanol producing ortholog expressed in ripe tomato fruit. Purple regions indicate complimentary PMEI and pectinesterase domains indicated by their Pfam designations. (DOCX 54 kb)

Additional file 6: Protocol S1. Synthesis of farnesyl acyl esters from farnesol and acid chlorides. (DOCX 14 kb)

## Abbreviations

1-MCP: 1-methylcyclopropene
AAT: Alcohol acyltransferase
AMDIS: Automated mass spectral deconvolution and identification software
ASG: Acylated steryl glycoside
CTOL: 2,6,10-trimethyldodeca-2,7(*E*),9(*E*),11-tetraen-6-ol
DPA: Diphenylamine
HPLC-APCI-MS: high performance liquid chromatography-atmospheric pressure chemical ionization-mass spectrometry; gas chromatography-mass spectrometry
HR: Hypersensitive response
MBPLS-DA: Multiblock partial least squares-discriminant analysis
MGDG: Monogalactosyldiglyceride
ORA: Overexpression analysis
PCA: Principal components analysis
PCD: Programmed cell death
PCR: Polymerase chain reaction
PLSR: Partial least squares regression
PME: Pectinmethylesterase
ROS: Reactive oxygen species
RPKM: Reads per kilobase of exon model per million mapped reads
TMS-oxime: Trimethylsilyl-oxime
VIP: Variables’ importance in the projection
