# Supplementary material for: Delayed response to cold stress is characterized by successive metabolic shifts culminating in apple fruit peel necrosis
Source: BMC Plant Biol. 2017 Apr 21;17:77. doi: 10.1186/s12870-017-1030-6 (PMC5399402; doi:10.1186/s12870-017-1030-6)
Supplement: Supplementary file 2 — Principal Components Analysis (PCA) bi-plots of metabolite (top) and gene expression level data (bottom) from ‘Granny Smith’ apple peel from fruit stored in air at 1 °C for up to 183 days (6 months). Apples were treated immediately following harvest with 2000 μL L−1 DPA or 1 mL L−1 1-MCP. Gene expression and metabolite datasets were analyzed separately. Shapes represent scores for each observation where symbol size increases with storage duration and symbol color represents scald severity. Gray points represent loadings for individual metabolites or transcripts. (DOCX 2373 kb) [file 12870_2017_1030_MOESM2_ESM.docx]

Scald severity (1-4)

Figure S1. Principal Components Analysis (PCA) bi-plots of metabolite (top) and gene expression level data (bottom) from ‘Granny Smith’ apple peel from fruit stored in air at 1 °C for up to 183 days (6 months). Apples were treated immediately following harvest with 2000 µL L^-1^ DPA or 1 mL L^-1^ 1-MCP. Gene expression and metabolite datasets were analyzed separately. Shapes represent scores for each observation where symbol size increases with storage duration and symbol color represents scald severity. Gray points represent loadings for individual metabolites or transcripts.
