# Supplementary material for: Delayed response to cold stress is characterized by successive metabolic shifts culminating in apple fruit peel necrosis
Source: BMC Plant Biol. 2017 Apr 21;17:77. doi: 10.1186/s12870-017-1030-6 (PMC5399402; doi:10.1186/s12870-017-1030-6)
Supplement: Supplementary file 3 — Gene cluster assignment for correlation networks. To summarize gene expression data, genes expression patterns from all time points (0–183 d) and treatments (control, diphenylamine, and 1-methylcyclopropene) were clustered using k-means clustering. Average gene expression values from each cluster were correlated along with relative metabolite levels to generate 2 month and 6 month networks. The number of Variables Important in the Projection (VIP) for the “scald severity” response variable from the PLS-DA model is included for each gene cluster. (DOCX 18 kb) [file 12870_2017_1030_MOESM3_ESM.docx]

Table S3. Gene cluster assignment for correlation networks. To summarize gene expression data, genes expression patterns from all time points (0-183 d) and treatments (control, diphenylamine, and 1-methylcyclopropene) were clustered using k-means clustering. Average gene expression values from each cluster were correlated along with relative metabolite levels to generate 2 month and 6 month networks. The number of Variables Important in the Projection (VIP) for the “scald severity” response variable from the PLS-DA model is included for each gene cluster. Clusters 7, 33, and 55 (emboldened) are first neighbors of methanol in the 6 month network.

| cluster | Genes/cluster | VIP  genes | cluster | Genes/cluster | VIP  genes | cluster | Genes/cluster | VIP genes |
| --- | --- | --- | --- | --- | --- | --- | --- | --- |
| 1 | 596 | 0 | 30 | 260 | 0 | 59 | 468 | 0 |
| 2 | 336 | 272 | 31 | 281 | 9 | 60 | 354 | 0 |
| 3 | 302 | 0 | 32 | 385 | 0 | 61 | 365 | 0 |
| 4 | 450 | 21 | ***33*** | ***1275*** | ***1206*** | 62 | 422 | 5 |
| 5 | 467 | 3 | 34 | 394 | 30 | 63 | 447 | 0 |
| 6 | 271 | 0 | 35 | 210 | 0 | 64 | 344 | 0 |
| ***7*** | ***371*** | ***262*** | 36 | 290 | 21 | 65 | 431 | 130 |
| 8 | 340 | 0 | 37 | 645 | 76 | 66 | 447 | 189 |
| 9 | 354 | 28 | 38 | 603 | 56 | 67 | 329 | 0 |
| 10 | 413 | 0 | 39 | 313 | 29 | 68 | 501 | 320 |
| 11 | 236 | 0 | 40 | 508 | 145 | 69 | 342 | 12 |
| 12 | 265 | 0 | 41 | 509 | 0 | 70 | 267 | 2 |
| 13 | 524 | 22 | 42 | 381 | 87 | 71 | 368 | 155 |
| 14 | 225 | 15 | 43 | 256 | 0 | 72 | 414 | 0 |
| 15 | 371 | 0 | 44 | 601 | 153 | 73 | 297 | 3 |
| 16 | 915 | 249 | 45 | 305 | 1 | 74 | 317 | 4 |
| 17 | 448 | 22 | 46 | 370 | 2 | 75 | 422 | 0 |
| 18 | 308 | 82 | 47 | 356 | 3 | 76 | 464 | 165 |
| 19 | 313 | 1 | 48 | 405 | 27 | 77 | 391 | 54 |
| 20 | 486 | 28 | 49 | 533 | 292 | 78 | 374 | 73 |
| 21 | 315 | 9 | 50 | 483 | 0 | 79 | 374 | 0 |
| 22 | 610 | 81 | 51 | 247 | 0 | 80 | 337 | 0 |
| 23 | 370 | 7 | 52 | 454 | 0 | 81 | 239 | 36 |
| 24 | 383 | 52 | 53 | 210 | 119 | 82 | 419 | 16 |
| 25 | 542 | 94 | 54 | 402 | 16 | 83 | 325 | 0 |
| 26 | 352 | 6 | ***55*** | ***460*** | ***338*** | 84 | 586 | 404 |
| 27 | 830 | 1 | 56 | 476 | 0 | 85 | 420 | 1 |
| 28 | 347 | 10 | 57 | 283 | 8 | 86 | 304 | 7 |
| 29 | 830 | 15 | 58 | 391 | 3 |  |  |  |
