# Supplementary material for: Delayed response to cold stress is characterized by successive metabolic shifts culminating in apple fruit peel necrosis
Source: BMC Plant Biol. 2017 Apr 21;17:77. doi: 10.1186/s12870-017-1030-6 (PMC5399402; doi:10.1186/s12870-017-1030-6)
Supplement: Supplementary file 4 — Comparative expression of scald VIPs and the “refined” methanol and CTOL subnetworks within different MapMan cell wall metabolism bins. Darker blue squares indicate elevated expression compared with the other lists within that category. (DOCX 29 kb) [file 12870_2017_1030_MOESM4_ESM.docx]

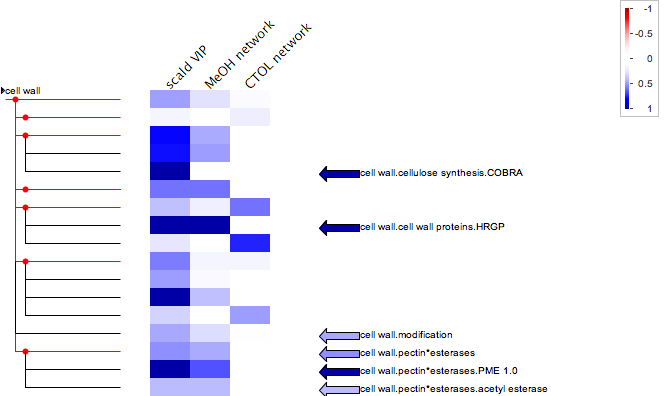


Figure S2. Comparative expression of scald VIPs and the “refined” methanol and CTOL subnetworks within different MapMan cell wall metabolism bins. Darker blue squares indicate elevated expression compared with the other lists within that category.
