# Supplementary material for: Delayed response to cold stress is characterized by successive metabolic shifts culminating in apple fruit peel necrosis
Source: BMC Plant Biol. 2017 Apr 21;17:77. doi: 10.1186/s12870-017-1030-6 (PMC5399402; doi:10.1186/s12870-017-1030-6)
Supplement: Supplementary file 5 — Alignment and domain comparison of scald-related MdPMEs, AT1G02810.1, and Solyc07g064170.2.1, a methanol producing ortholog expressed in ripe tomato fruit. Purple regions indicate complimentary PMEI and pectinesterase domains indicated by their Pfam designations. (DOCX 54 kb) [file 12870_2017_1030_MOESM5_ESM.docx]

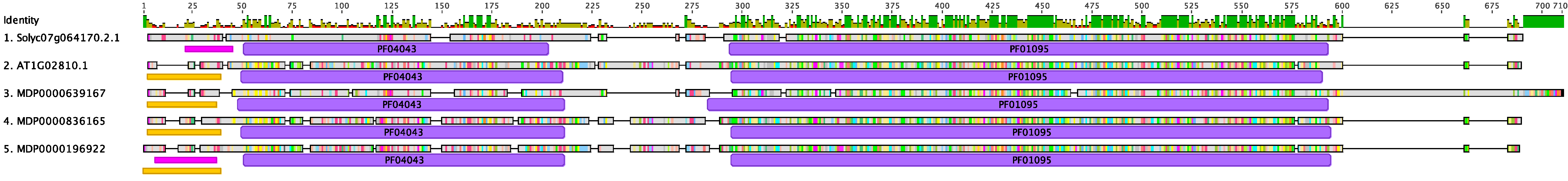


Figure S3. Alignment and domain comparison of scald-related MdPMEs, AT1G02810.1, and Solyc07g064170.2.1, a methanol producing ortholog expressed in ripe tomato fruit. Purple regions indicate complimentary PMEI and pectinesterase domains indicated by their Pfam designations.
