# Supplementary material for: Delayed response to cold stress is characterized by successive metabolic shifts culminating in apple fruit peel necrosis
Source: BMC Plant Biol. 2017 Apr 21;17:77. doi: 10.1186/s12870-017-1030-6 (PMC5399402; doi:10.1186/s12870-017-1030-6)
Supplement: Supplementary file 6 — Synthesis of farnesyl acyl esters from farnesol and acid chlorides. (DOCX 14 kb) [file 12870_2017_1030_MOESM6_ESM.docx]

**Protocol S1** Synthesis of farnesyl acyl esters from farnesol and acid chlorides.

**Farnesyl oleate, farnesyl linolenate, and farnesyl linoleate standard synthesis**

10 μL farnesol (*l*) was placed into a 2 ml vacuum vial. Then 24 µl acyl chloride (oleic chloride, linolenic chloride, or linoleic chloride), 10 µl pyridine, and 1 ml of xylene were added to the farnesol. The vial was then vacuum sealed, and heated for 1 h at 200°C.
